# Opportunities to improve detection of genetic predisposition for ovarian cancer applying the Tumor-First workflow

**DOI:** 10.1007/s10689-026-00592-x

**Published:** 2026-08-04

**Authors:** Vera M. Witjes, Joanne A. de Hullu, Angela van Remortele, Lilian Vreede, Michiel Simons, Nicoline Hoogerbrugge, Marjolijn J. L. Ligtenberg

**Affiliations:** 1 Department of Human Genetics, Radboud university medical center, Nijmegen, The Netherlands; 2 Research Institute for Medical Innovation, Radboud university medical center, Nijmegen, The Netherlands; 3 Department of Obstetrics and Gynecology, Radboud university medical center, Nijmegen, The Netherlands; 4 Department of Pathology, Radboud university medical center, Nijmegen, The Netherlands

**Keywords:** Ovarian cancer, Genetic testing, Hereditary cancer, Tumor DNA, BRCA, Germline

## Abstract

Genetic testing in ovarian carcinoma (OC) patients is very important for patients and their relatives. The Tumor-First workflow uses a tumor DNA test to stratify germline testing for hereditary cancer predisposition as well as treatment options with PARP inhibitors. This workflow is adopted and successfully implemented nationwide. Here, we evaluated recent tumor DNA testing rates in the Netherlands to identify untested OC patient groups and optimize tumor DNA testing rates. OC patients diagnosed in 2023 or 2024 were selected from the Netherlands Cancer Registry. We analyzed patient characteristics associated with the likelihood of tumor DNA testing using multivariable logistic regression. Tumor-First testing was performed for 1765 out of the 2221 (79%) OC patients. Patients diagnosed with advanced stage OC were more likely to receive tumor DNA testing (OR = 2.5, *p* < 0.001) than those with low-stage disease. Compared with patients who underwent surgery as primary treatment, those who received chemotherapy or no primary treatment were less likely to be tested (OR = 0.21 and OR = 0.07, respectively, *p* < 0.001). Patients who died within 100 days were less likely to receive testing (OR = 0.57, *p* = 0.003). Patients without resection after diagnosis on biopsy/cytology had the lowest Tumor-First testing rates (58% vs. 87–95%, χ2 *p* < 0.001). Tumor-First testing rates are high. However, OC patients that do not undergo a resection are less likely to undergo testing. With these data strategies to identify hereditary cancer predisposition in patients and their relatives can be further improved.

**Supplementary Information:**

The online version contains supplementary material available at 10.1007/s10689-026-00592-x.

## Introduction

Genetic testing in ovarian carcinoma (OC, i.e., ovarian, tubal and primary peritoneal carcinoma) can save lives. Germline testing is important to detect a hereditary cancer predisposition, i.e., a germline (likely) pathogenic variant (PV) in *BRCA1*, *BRCA2*, *RAD51C*, *RAD51D*, *BRIP1* or *PALB2*, which can be lifesaving in relatives by surveillance and/or preventive surgery. Tumor DNA testing is important because OC patients with both germline and somatic *BRCA1/2* PVs can benefit from additional PARP inhibitor therapy [1].

The Tumor-First workflow for genetic testing in OC was developed a decade ago and later adopted and implemented in all centers in the Netherlands [2–4]. With this workflow, a primary tumor DNA test is used to stratify patient eligibility for hereditary cancer testing as well as PARP inhibitor treatment. Germline testing is offered to patients with a PV in an OC risk gene identified in tumor DNA, as well as to patients with a family history suggestive of hereditary OC and patients with an unsuccessful tumor DNA test. The Tumor-First workflow has shown to be a patient-friendly and cost-effective alternative to the traditional referral-based genetic testing of all OC patients [5].

The Tumor-First workflow was implemented very successfully nationwide. Over the years, the testing rates amongst patients with a resected OC increased from 50 to 85% [6]. To further optimize the Tumor-First workflow, investigation into tumor DNA testing rates across the entire population of OC patients, including those without a resection, was warranted.

Although the Tumor-First workflow aims to test all OC, we hypothesize that clinical and pathological factors influence whether tumor DNA testing occurs. The pathologist performs the initial step in the workflow with reflex testing of all OC, but the ultimate responsibility of test initiation lies within the multidisciplinary treatment team. Patients with limited contact with this team may be less likely to be tested. Additionally, because tumor DNA results guide PARP‑inhibitor eligibility, patients whose histology and grade do not qualify them for PARP inhibitors may also be tested less often. The aim of the current study is to evaluate the nationwide tumor DNA testing rates and to explore how these relate to clinical and pathological characteristics of OC patients.

## Methods

This is a retrospective observational study investigating the nationwide tumor DNA testing rates in OC. The local medical ethics committee of the Radboudumc assessed that this evaluation was not subject to the Medical Research Involving Human Subjects Act (WMO) (Number 2024–17668).

### Data collection

Adult patients with OC, i.e., ovarian, tubal and primary peritoneal carcinoma, diagnosed in 2023 or 2024, were selected from the Netherlands Cancer Registry (NCR). The NCR establishes OC diagnoses using information from pathology reports and clinical data extracted from medical records. All OC patients that were registered at the time of data collection (April 2025) were included in the study. Registration for patients diagnosed in 2024 was not yet complete at that timepoint, particularly those diagnosed in the last quarter of 2024. Clinical patient characteristics were obtained from the NCR and pathology reports were retrieved from the nationwide pathology databank (Palga) [7]. OC pathology reports, updated until April 2025, were reviewed to extract data on OC diagnosis and information on tumor DNA testing, such as the date, tissue type and PV detection. The four-month interval between case selection and pathology report collection should allow tumor DNA testing to be completed, minimizing potential bias in testing rates.

### Data analysis

The main outcome of the study was the rate of tumor DNA testing, hereafter referred to as Tumor-First testing, and the association with clinical and pathological patient characteristics. Patient population characteristics were summarized as median and interquartile range (IQR) for numerical variables, and number and percentages for categorical variables. Factors influencing Tumor-First testing rates were evaluated with univariate and multivariable logistic regression.

Factors included in the model were selected based on our hypothesis and related to the amount of contact with the multidisciplinary treatment team and PARP inhibitor treatment eligibility. Patient age, primary tumor-directed therapy (i.e., the most important treatment being either surgery, chemotherapy or other), and death within 100 days of diagnosis are factors indicating the amount of contact with the multidisciplinary treatment team. Tumor type, tumor FIGO stage, and year of diagnosis are factors indicating PARP inhibitor treatment eligibility. Year of diagnosis is important in this regard because HRD testing was implemented within the workflow in 2024 as an additional biomarker for PARP inhibitor treatment effectiveness [8].

The same model and variables as those used in the analysis of tumor DNA testing rates were included to assess factors associated with OC risk gene PV detection rates, allowing comparison between factors influencing testing and PV detection. Overall group differences were compared using a chi-square test. Two-sided *p*-values < 0.05 were considered statistically significant. Analyses were performed in R (v4.4.1) using RStudio (2024.04).

## Results

### Population description and general characteristics

In total, 2327 patients diagnosed with OC in 2023 or 2024 were identified through the NCR. Pathology reports were obtained for 2221 patients diagnosed with OC, and these patients were included in the study. For 89 patients, pathology data were unavailable due to a clinical diagnosis only, and for 17 patients pathology data were missing for unknown reasons. Therefore, at most 106 patients registered with OC (5%) do not have a pathologically confirmed diagnosis.

The general characteristics of the patient population that is pathologically confirmed are provided in Table 1. The median age of the patients was 69 years, and 57% had high-grade serous OC. Most patients were diagnosed with stage III or stage IV tumors, 38 and 36% respectively. 1913 patients (86%) received tumor-directed therapy, and surgery was the main treatment approach for most patients (78%). 290 patients (13%) died within 100 days after the OC diagnosis.

**Table 1:** General characteristics and Tumor-First testing characteristics of the OC patients with a pathological assessment

Age—M (IQR), N = 2221		69 (60–76)
Year of diagnosis, N = 2221	2023	1244 (56.0%)
	2024	977 (44.0%)
Tumor histology, N = 2221	High-grade serous	1264 (56.9%)
	Mucinous	166 (7.5%)
	Clear cell	141 (6.3%)
	Endometrioid	135 (6.1%)
	Low-grade serous	106 (4.8%)
	Carcinosarcoma	39 (1.8%)
	Other	30 (1.4%)
	Serous, NOS	110 (5.0%)
	(Adeno)carcinoma, NOS	230 (10.4%)
FIGO, N = 2221	Stage I	383 (17.2%)
	Stage II	178 (8.0%)
	Stage III	840 (37.8%)
	Stage IV	794 (35.7%)
	Unknown	26 (1.2%)
Tumor-directed therapy, N = 2221	Yes	1913 (86.1%)
	No	305 (13.7%)
	Unknown	3 (0.1%)
Type of primary therapy, N = 1913	Surgery	1486 (77.7%)
	Chemotherapy	366 (19.1%)
	Targeted therapy	39 (2.0%)
	Hormonal therapy	22 (1.2%)
Early-mortality, N = 2221	Alive 100 days after diagnosis	1931 (86.9%)
	Died < 100 days after diagnosis	290 (13.1%)
Tumor-First performed, N = 2221	Yes	1765 (79.5%)
	No	456 (20.5%)
Tumor-First successful, N = 1765	Yes	1666 (94.4%)
	No	99 (5.6%)
Tumor-First result, N = 1666	No PV identified	1416 (85.0%)
	PV identified	250 (15.0%)
Gene PV, N = 250	*BRCA1*	141 (56.4%)
	*BRCA2*	80 (32.0%)
	*RAD51C*	11 (4.4%)
	*BRIP1*	5 (2.0%)
	*PALB2*	5 (2.0%)
	*RAD51D*	4 (1.6%)
	Multiple genes*	4 (1.6%)

^*^BRCA2 & *RAD51C* (n = 2), *BRCA1* & *BRCA2* (n = 1), *BRCA1* & *RAD51D* (n = 1). 4 patients had a 2nd primary tumor

NOS, not otherwise specified, PV , (likely) pathogenic variant

Tumor-First testing was performed for 1765 out of the 2,221 (79%) patients. 1666 (94%) of these Tumor-First tests were successful and in 250 (15%) a PV in an OC risk gene was detected, the majority of which in *BRCA1* (n = 141) or *BRCA2* (n = 80).

### Factors associated with Tumor-First test uptake

The likelihood of the Tumor-First test being performed differed across the clinical patient characteristics, as presented in Table 2. In the multivariable model including all the variables, several factors were significantly associated with Tumor-First test uptake.

**Table 2:** Odds ratios of the likelihood of Tumor-First testing across various clinical characteristics from univariate and multivariable logistic regression

Variable	Category	Total	Tested TF	Univariate		Multivariable	
		*N*	*n (%)*	*OR (95% CI)*	*P-value*	*OR (95% CI)*	*P-value*
Year of diagnosis	2023 (ref)	1244	965 (77.6)	1.00		1.00	
	2024	977	800 (81.9)	1.31 (1.06–1.61)	0.013	1.43 (1.12–1.84)	0.005
Histology	High-grade serous (ref)	1264	1074 (85)	1.00		1.00	
	Non-high-grade serous^*^	617	522 (84.6)	0.97 (0.75–1.27)	0.836	0.80 (0.56–1.15)	0.225
	Serous NOS	110	80 (72.7)	0.47 (0.30–0.75)	0.001	0.79 (0.47–1.36)	0.375
	(Adeno)carcinoma NOS	230	89 (38.7)	0.11 (0.08–0.15)	< 0.001	0.35 (0.24–0.50)	< 0.001
FIGO	Stage I/II (ref)	561	470 (83.8)	1.00		1.00	
	Stage III/IV	1634	1285 (78.6)	0.71 (0.55–0.92)	0.009	2.52 (1.70–3.77)	< 0.001
Main tumor-directed therapy	Surgery (ref)	1486	1346 (90.6)	1.00		1.00	
	Chemotherapy	366	267 (73)	0.28 (0.21–0.38)	< 0.001	0.21 (0.14–0.31)	< 0.001
	Other therapy	61	46 (75.4)	0.32 (0.18–0.6)	< 0.001	0.24 (0.12–0.48)	< 0.001
	No therapy	305	105 (34.4)	0.06 (0.04–0.07)	< 0.001	0.07 (0.04–0.11)	< 0.001
Early mortality	Alive 100 days after diagnosis (ref)	1931	1653 (85.6)	1.00		1.00	
	Died < 100 days after diagnosis	290	112 (38.6)	0.11 (0.08–0.14)	< 0.001	0.57 (0.39–0.83)	0.003
Age groups	65–75 (ref)	700	585 (83.6)	1.00		1.00	
	< 35	36	34 (94.4)	3.34 (1.00–20.77)	0.101	2.29 (0.66–14.52)	0.269
	35–45	78	68 (87.2)	1.34 (0.70–2.83)	0.412	0.81 (0.40–1.79)	0.578
	45–55	230	192 (83.5)	0.99 (0.67–1.50)	0.974	0.74 (0.47–1.20)	0.214
	55–65	476	412 (86.6)	1.26 (0.91–1.77)	0.163	0.97 (0.67–1.41)	0.864
	75–85	595	437 (73.4)	0.54 (0.41–0.71)	< 0.001	1.03 (0.75–1.42)	0.851
	> 85	106	37 (34.9)	0.10 (0.07–0.16)	< 0.001	0.54 (0.31–0.93)	0.027

FIGO stage unknown (n = 26), an tumor-directed therapy unknown (n = 3) are excluded from these analyses

^*^including mucinous, clear cell, endometrioid, low-grade serous, carcinosarcomas and other histological subtypes

NOS , not otherwise specified; TF , tumor-first; ref, reference

Patients diagnosed in 2024 were more likely to receive testing compared to patients diagnosed in 2023 (OR = 1.4, *p* = 0.005). Also, patients diagnosed with stage III or IV tumors were more likely to receive testing compared to those with stage I or II tumors (OR = 2.5, *p* < 0.001). Compared with patients whose main treatment was surgery, patients whose main treatment was chemotherapy or who received no treatment were less likely to be tested (OR = 0.21 and OR = 0.07, respectively, *p* < 0.001). Furthermore, patients who died shortly after diagnoses, within 100 days, were less likely to receive testing (OR = 0.57, *p* = 0.003), similar to the oldest age group of > 85 years (OR = 0.54, *p* = 0.027). The test percentages did not differ between the high-grade serous OC group and the other specified histological subtypes, but the adenocarcinoma NOS group was less likely to receive testing than the high-grade serous group (OR = 0.35, *p* < 0.001).

The likelihood of PV detection also varied across the variables (supplementary Table 1). In the multivariable model, patients with non-high-grade serous OC were less likely to have a PV in an OC risk gene (OR = 0.08, *p* < 0.001). Patients who had chemotherapy as primary treatment were less likely to have a PV than those treated primarily with surgery (OR = 0.35, *p* < 0.001), while no significant difference was observed between surgery and no treatment. Additionally, younger patients were more likely to have a PV compared to the median age group, with the highest odds in the 45–55 year old patient group (OR = 3.4, *p* < 0.001). No significant differences were observed for the year of diagnosis and tumor stage.

### Type of tissue material: frequency and timelines of testing

**Fig. 1:**
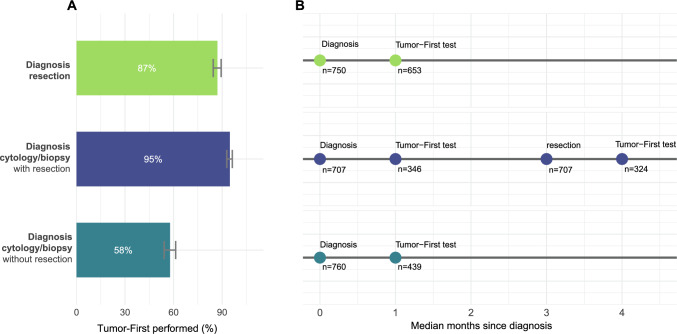
**A** Stacked bar-chart of Tumor-First testing rates across groups defined by tissue type of diagnosis. Error bars represent the 95% confidence intervals. **B** Timelines of Tumor-First testing across the three groups with the median months between events and including the numbers tested. *Notes:* Material diagnoses unknown (n = 4) are excluded from this figure

Figure 1A presents the variation in Tumor-First testing rates among the types of tissue material in the pathology report at diagnosis, which was reported for 2217 out of the 2221 patients. We defined three groups: (1) for 750 patients (39%) the first pathology assessment was on resected tumor tissue, (2) for 707 patients (32%) the first pathology report was cytology or biopsy with follow-up of a resection, and (3) for 760 patients (34%) biopsy or cytology was not followed-up with a resection. These groups varied significantly in Tumor-First testing percentages (87, 95 and 58% respectively, χ2 *p* < 0.001). In the group of patients that were diagnosed based on a biopsy or cytology with a follow-up of resection, 346 out of the 670 (52%) tests were performed before the resection, and 324 after (48%). For all groups together, the median time between diagnosis and Tumor-First test result was 31 days (IQR 30—90). The timelines of testing for the specified patient groups are presented in Fig. 1B.

## Discussion

Although the overall Tumor-First testing rates are high (79%), we highlight specific OC patient groups who are at risk of not receiving such testing. OC patients who did not receive tumor-directed therapy, patients with stage I or II tumors, patients with a tumor morphology of adenocarcinoma NOS, patients who died within 100 days after diagnosis, and patients in the oldest age group were less likely to receive Tumor-First testing. Similarly, the group of OC patients where the first pathology report included cytology or biopsy material without follow-up of a resection, was least likely to receive Tumor-First testing. This knowledge can help to develop strategies to improve genetic testing rates. This is very relevant as identification of OC predisposition is important for relatives of all OC.

The tumor DNA testing rate of 79% is high compared with germline testing rates reported in large observational studies over the previous decade, about 35–40% [9, 10]. Despite being only the initial step of the workflow, and therefore not directly comparable to germline testing rates, this high tumor DNA testing rate plays a key role in the Tumor-First workflow and contributes to the detection of a genetic predisposition in OC. Nevertheless, consistent with the findings of Lau-Min et al., who investigated the uptake of germline testing in a nationwide cohort [9], we found that patients with low-stage tumors are less likely to receive testing. This observation may be explained by the restriction of PARP inhibitors to advanced-stage disease. Treatment indications might have further influenced the Tumor-First testing uptake as patients diagnosed in 2024 were more likely to be tested. This increase may be attributed to the implementation of routine HRD testing in the Netherlands in 2024 to determine eligibility for first-line maintenance treatment with niraparib [8]. Our data indicates that, although the Tumor-First protocol advises that the pathologist initiates testing upon the first diagnosis for all OC patients, testing is often postponed until resection. Reasons for this may be that material available at diagnosis may be unsuitable for tumor DNA testing, for example due to a limited amount of tissue or a low tumor cell percentage, or that the diagnosis may be uncertain at that timepoint.

On the other hand, in contrast to previous studies reporting higher testing rates in high-grade serous OC [11, 12], we did not observe a difference in tumor DNA testing rates between the high-grade serous OC compared to non-high-grade serous histological subtypes. The findings in other studies can be explained by the higher prevalence of *BRCA1/2* PV in the high-grade serous OC group [13], which we also observed in the current study. We did observe that the patients with a tumor morphology of adenocarcinoma not otherwise specified were less likely to be tested. In these cases with unclear histology, the diagnosis of OC may have been uncertain at the time of pathology review and may later have been established based on clinical features. Awareness amongst clinicians to request DNA testing for this specific patient population is important.

Other groups of patients that should receive attention include those who do not receive treatment, die shortly after diagnosis, or are of advanced age, with likely overlap between these subgroups. While we did not observe a difference in PV detection rates, our data suggests that patients who solely get palliative care following their OC diagnosis are less likely to receive tumor DNA testing. This implies that patients who are less closely monitored by a gynecological oncologist and/or do not need tumor testing for therapy stratification, are less likely to be tested. While the tumor DNA test result may not have clinical implications for the patient, it remains essential for relatives as it identifies whether a hereditary genetic aberration may be present and requires further evaluation. Therefore, awareness in multidisciplinary oncology meetings remains of critical importance to stimulate the use of the Tumor-First test or to stimulate germline DNA testing in case tumor DNA testing is not expected to be possible in the near future.

A strong aspect of this study is the use of a nationwide representative dataset with findings that reflect routine clinical practice allowing us to identify areas for improvement of care. A key limitation is the absence of data on PARP‑inhibitor effectiveness and germline testing outcomes. While the tumor DNA testing rates offer valuable insight into the initial step of the Tumor-First workflow, they do not allow evaluation of the full diagnostic pathway and clinical outcomes. Another limitation is that our study does not provide detail at the patient level, and the exact reasons for not receiving tumor DNA testing per individual patient remain unknown. Additionally, we excluded the population of OC patients who are diagnosed clinically without tissue-based confirmation from the current study, as they cannot receive tumor DNA testing. Nevertheless, genetic testing is important for this population and they should be offered germline testing. Although the uptake of germline testing in this population remains unknown, these OC patients, who are likely provided palliative care following diagnosis, are at risk of missing out on genetic testing, as referral for genetic counseling is often considered too burdensome for patients in this condition. Initiation of germline genetic testing by non-genetic healthcare professionals may offer a way to reach these patients, as it has been proven effective in improving germline testing rates [14]. Alternatively, securing a blood sample can be a useful approach to enable future genetic testing for at-risk relatives.

Despite advocacy for a Tumor-First workflow for OC by groups in several other countries [15–17], large‑scale implementation has, to our knowledge, not been achieved elsewhere. In our previous study, we have shown that implementation of the Tumor-First workflow was effective at a nationwide level in the Netherlands, thereby improving the detection of genetic predisposition among OC patients and their at-risk relatives [6]. Nevertheless, with this study, we highlighted specific patient groups who are at risk of not receiving testing. By stimulating tumor DNA testing on cytological and biopsy materials, and initiating a germline DNA test in case no surgery is foreseen, we can further improve the detection of a genetic predisposition in OC. This increases opportunities for targeted treatment in patients and for cancer prevention in their relatives, which may save lives.

## Supplementary Information

Below is the link to the electronic supplementary material.

Supplementary file1

## Data Availability

The data supporting this article are available upon reasonable request.
