# Supplementary material for: Opportunities to improve detection of genetic predisposition for ovarian cancer applying the Tumor-First workflow
Source: Fam Cancer. 2026 Aug 4;25(3):82. doi: 10.1007/s10689-026-00592-x (PMC13437641; doi:10.1007/s10689-026-00592-x)
Supplement: Supplementary file 1 [file 10689_2026_592_MOESM1_ESM.pdf]

Supplementary information

Opportunities to improve detection of genetic predisposition for ovarian cancer applying the Tumor-First workflow

Vera M. Witjes, Joanne A. de Hullu, Angela van Remortele, Lilian Vreede, Michiel Simons, Nicoline Hoogerbrugge, Marjolijn J.L. Ligtenberg

Familial Cancer, 2026

Supplementary Table 1. Odds ratios of the likelihood of PV detection in successful Tumor-First tests across various clinical characteristics from univariate and multivariate logistic regression.

**Variable**	**Category**	**Total**	**PV detected**	**Univariate**		**Multivariate**	
		N	*n (%)*	*OR (95% CI)*	*P-value*	*OR (95% CI)*	*P-value*
**Year of diagnosis**	2023 (ref)	923	142 (15.4%)	1.00		1.00	
	2024	743	108 (14.5%)	0.94 (0.71–1.23)	0.630	0.98 (0.73–1.32)	0.918
**Histology**	High-grade serous (ref)	1028	218 (21.2%)	1.00		1.00	
	Non-high-grade serous*	487	16 (3.3%)	0.13 (0.07–0.21)	<0.001	0.08 (0.04–0.14)	<0.001
	Serous NOS	75	9 (12%)	0.51 (0.23–0.98)	0.061	0.57 (0.24–1.17)	0.154
	(Adeno)carcinoma NOS	76	7 (9.2%)	0.38 (0.16–0.78)	0.016	0.69 (0.27–1.56)	0.400
**FIGO**	Stage I/II (ref)	442	38 (8.6%)	1.00		1.00	
	Stage III/IV	1215	209 (17.2%)	2.21 (1.55–3.22)	<0.001	1.09 (0.72–1.69)	0.690
**Main tumor-directed therapy**	Surgery (ref)	1283	209 (16.3%)	1.00		1.00	
	Chemotherapy	239	16 (6.7%)	0.37 (0.21–0.61)	<0.001	0.35 (0.19–0.6)	<0.001
	Other therapy	45	18 (40%)	3.43 (1.82–6.29)	<0.001	3.90 (1.98–7.62)	<0.001
	No therapy	99	7 (7.1%)	0.39 (0.16–0.8)	0.019	0.55 (0.19–1.41)	0.240
**Early mortality**	Alive 100 days after diagnosis (ref)	1561	243 (15.6%)	1.00		1.00	
	Died <100 days after diagnosis	105	7 (6.7%)	0.39 (0.16–0.79)	0.017	0.75 (0.26–2.00)	0.586
**Age groups**	65-75 (ref)	553	79 (14.3%)	1.00		1.00	
	<35	32	2 (6.2%)	0.40 (0.06–1.36)	0.216	3.01 (0.44–12.17)	0.172
	35-45	66	10 (15.2%)	1.07 (0.50–2.10)	0.850	2.05 (0.90–4.37)	0.072
	45-55	182	43 (23.6%)	1.86 (1.22–2.81)	0.004	3.37 (2.10–5.40)	<0.001
	55-65	392	72 (18.4%)	1.35 (0.95–1.92)	0.092	1.7 (1.17–2.46)	0.005
	75-85	408	42 (10.3%)	0.69 (0.46–1.02)	0.066	0.78 (0.50–1.19)	0.250
	>85	33	2 (6.1%)	0.39 (0.06–1.31)	0.199	0.4 (0.06–1.60)	0.255

Notes: FIGO stage unknown (n =26), an tumor-directed therapy unknown (n=3) are excluded from these analyses.

[*] including mucinous, clear cell, endometrioid, low-grade serous, carcinosarcomas and other histological subtypes

Abbreviations: NOS = not otherwise specified, TF = Tumor-First, PV = (likely) pathogenic variant
